# A First for Human Vaccinology: GMP Compliant Radiation Attenuation of *Plasmodium falciparum* Sporozoites for Production of a Vaccine Against Malaria

**DOI:** 10.3389/fimmu.2022.851028

**Published:** 2022-02-15

**Authors:** Eric R. James, Steve Matheny, James Overby, B. Kim Lee Sim, Abraham G. Eappen, Tao Li, Ming Lin Li, Thomas L. Richie, Sumana Chakravarty, Anusha Gunasekera, Tooba Murshedkar, Peter F. Billingsley, Stephen L. Hoffman

**Affiliations:** Sanaria Inc., Rockville, MD, United States

**Keywords:** radiation, attenuation, malaria, sporozoite, vaccine

## Abstract

Ionizing radiation (UV, X-ray and ɣ) administered at an appropriate dose to pathogenic organisms can prevent replication while preserving metabolic activity. We have established the GMP process for attenuation by ionizing radiation of the *Plasmodium falciparum* (Pf) sporozoites (SPZ) in Sanaria^®^ PfSPZ Vaccine, a protective vaccine against malaria. Mosquitoes raised and infected aseptically with Pf were transferred into infected mosquito transport containers (IMTC) and ɣ-irradiated using a ^60^Co source. PfSPZ were then extracted, purified, vialed, and cryopreserved. To establish the appropriate radiation conditions, the irradiation field inside the IMTCs was mapped using radiochromic film and alanine transfer dosimeters. Dosimeters were irradiated for times calculated to provide 120-170 Gy at the minimum dose location inside the IMTC and regression analysis was used to determine the time required to achieve a lower 95% confidence interval for 150 Gy. A formula incorporating the half-life of ^60^Co was then used to construct tables of irradiation times for each calendar day. From the mapping studies, formulae were derived to estimate the minimum and maximum doses of irradiation received inside the IMTC from a reference dosimeter mounted on the outside wall. For PfSPZ Vaccine manufacture a dose of 150 Gy was targeted for each irradiation event, a dose known to completely attenuate PfSPZ. The reference dosimeters were processed by the National Institute of Standards and Technology. There have been 587 irradiation events to produce PfSPZ Vaccine during 13 years which generated multiple lots released for pre-clinical studies and clinical trials. The estimated doses at the minimum dose location (mean 154.3 ± 1.77 Gy; range 150.0-159.3 Gy), and maximum dose location (mean 166.3 ± 3.65 Gy, range 155.7 to 175.3 Gy), in IMTCs were normally distributed. Overall dose uniformity was 1.078 ± 0.012. There was no siginifcant change in measured dose over 13 years. As of January 2022, 21 clinical trials of PfSPZ Vaccine have been conducted, with 1,740 volunteers aged 5 months to 61 years receiving 5,648 doses of PfSPZ Vaccine totalling >5.3 billion PfSPZ administered. There have been no breakthrough infections, confirming the consistency and robustness of the radiation attenuation process.

## Introduction

Radiation wavelengths shorter than ~124 nm that include far-UV, X-ray and ɣ, induce ionization effects that damage live cells principally through the generation of free radicals and their interaction with proteins, membranes and DNA. The dose of radiation can be selected to render cells or whole organisms metabolically active but incapable of replication. Used on eukaryotic pathogens, irradiation is an ideal method for developing live attenuated vaccines that are immunogenic and for which the ability to cause disease has been abrogated. Ionizing radiation of all three types has been used to attenuate parasitic protozoa and helminths (1–9)^1^.

The pioneering studies on attenuation of malaria sporozoites (SPZ) for assessing protective immunity were conducted with X-ray as the irradiation source (10). Subsequent studies used X-rays and ɣ irradiation, sourced either from ^137^Cs or ^60^Co (11–14). Sanaria^®^PfSPZ Vaccine is composed of SPZ, the infective stage of *Plasmodium falciparum* (Pf), that are irradiated in the mosquito using a ^60^Co source and subsequently extracted from the mosquito salivary glands, purified, formulated with cryoprotectant additives and cryopreserved (15, 16). In clinical trials, PfSPZ Vaccine induces >90% protection against controlled human malaria infection (CHMI) delivered by mosquito bite or by injection (17–19) and significant protection for at least two malaria transmission seasons against natural exposure to malaria in Africa (20). Attenuation by ɣ irradiation was adopted for the manufacture of PfSPZ Vaccine principally due to the ability to deliver a very accurate irradiation dose, shorter irradiation times than X-ray, ease of use, and a history of success in human trials (14) that used PfSPZ administered by the bite of Pf-infected, irradiated mosquitoes for immunizations.

We present here the process for development of a robust and reproducible method for the ɣ-irradiation of PfSPZ-infected mosquitoes delivered by a ^60^Co source, and the experience of using this method in the manufacture of PfSPZ Vaccine for clinical trials.

## Materials and Methods

### Irradiator

**Figure 1:**
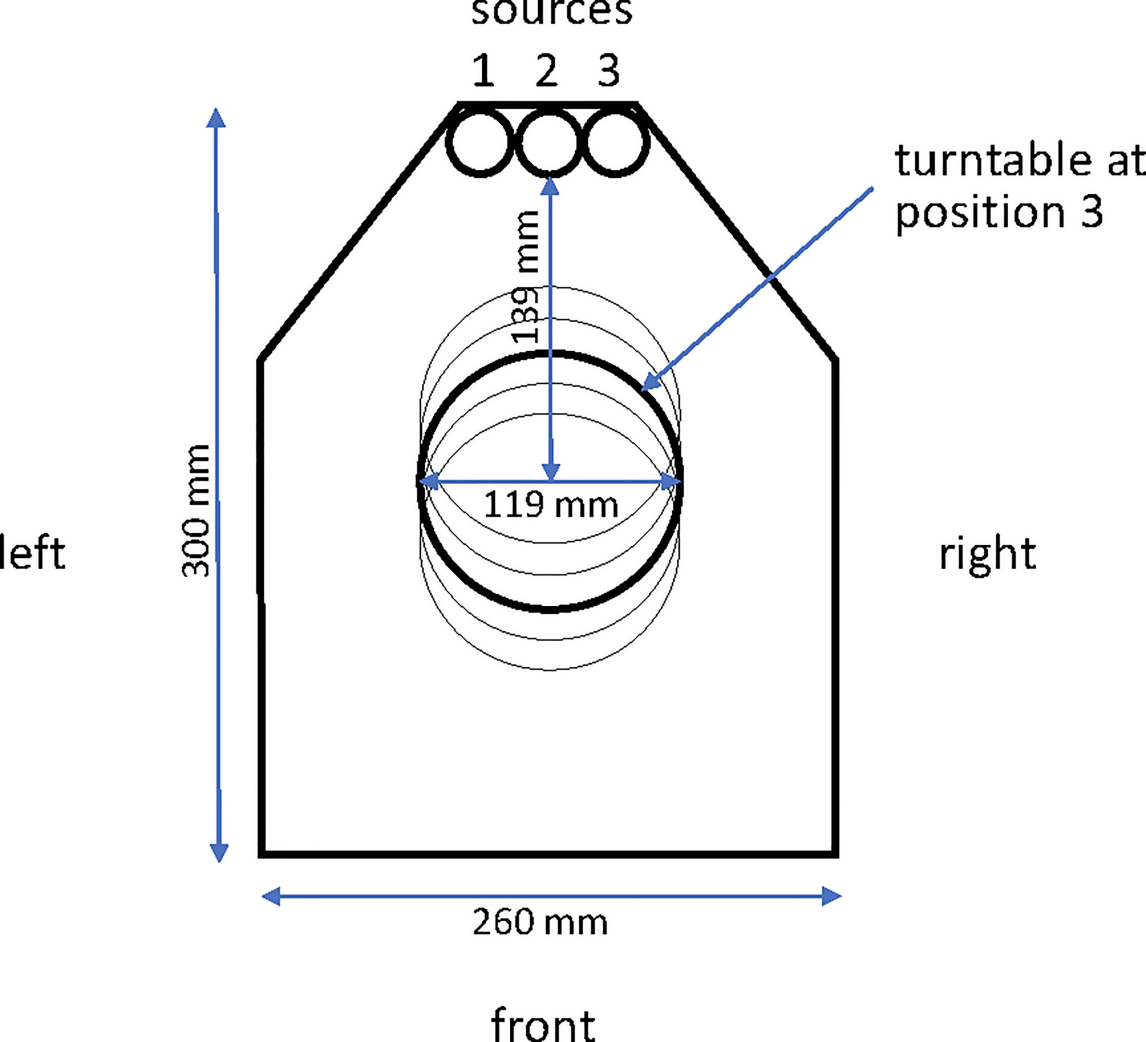
Plan view of the interior of the irradiator chamber. The three source tunnels (left #1, center #2 and right #3) are indicated at the apex of the drawing and the turntable is indicated at position 3 (of 5 potential positions) in the central axis of the chamber.

The irradiator, a JL Shepherd model 484-R-2, has three sources, an integral controller incorporating a timer and an air compressor with reservoir. The unit is fabricated principally from cast iron and lead, weighs approximately 6.5 metric tons, and houses the shielded irradiation chamber (**Figure 1**). The unit is calibrated annually by JL Shepherd (San Fernando, CA), and the controller and monitoring systems are calibrated independently every six months.

When installed in 2007, source tunnels 2 (center) and 3 (right) contained cobalt capsules with a total activity of 12,000 Ci. Nine years later, the ^60^Co had decayed through 1.9 half lives to 3,349 Ci so the ^60^Co capsule occupying source tunnel 2 was moved into source tunnel 1, and a new capsule with 8,400 Ci activity was added to source tunnel 2 to bring the total activity to 11,749 Ci. Sources are registered with the Nuclear Regulatory Commission and checked annually. In addition to the three source tunnels, the irradiator chamber contains a turntable that rotates at approximately 17 revolutions per minute and can be used at any of five positions at different distances from the irradiation sources. Turntable position 3 is used for IMTC mosquito irradiation (**Figure 1**).

Personnel qualified to operate the irradiator undergo FBI background checks, fingerprinting, and are issued a unique coded card for entry. Other security measures include a second coded door entry, a third door linked to an iris scanner, video surveillance at multiple locations and direct real-time video feed to the County Police Department (CPD) with an on-call Special Weapons and Tactics (SWAT) team.

Residual radiation around the irradiator both at rest and when active is equivalent to background, as indicated by routine dosimetry (processed quarterly) from multiple locations in the irradiator room. However, Sanaria provides operators with personal dosimeters that are maintained by a contract Radiation Safety Officer and processed by Landauer (Beltsville, MD). A survey meter (Technical Associates, Canoga Park, CA) connected to the irradiator controller broadcasts an alarm internally and to the CPD if radiation levels exceed threshold for safety or if the meter is disconnected or disabled. Additional monitoring and alarm systems are integral to the unit.

## Infected Mosquito Transport Container (IMTC)

**Figure 2:**
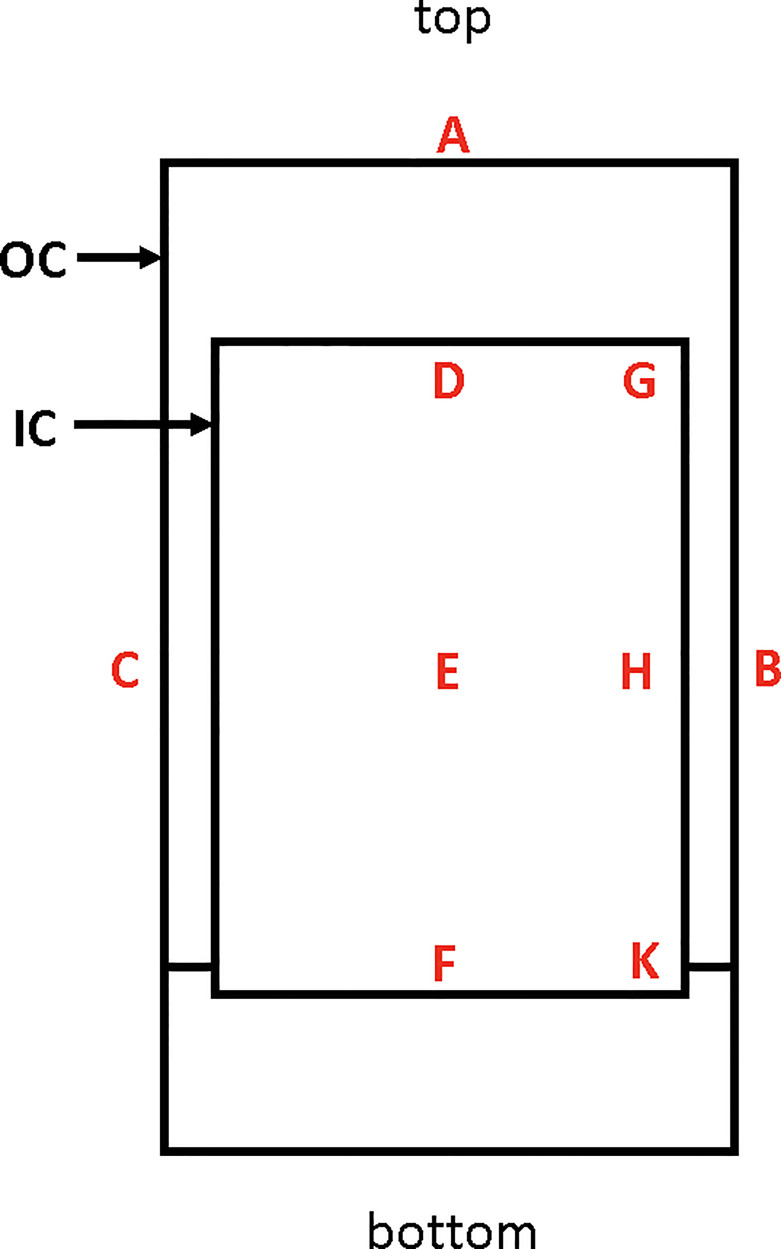
Sagittal sectional schematic diagram of an IMTC. The inner container (IC) is housed inside the outer container (OC), which is a custom machined 1 L polycarbonate container with screw top and screw base. All nine locations used for placement of alanine transfer dosimeters for mapping are indicated. The minimum dose of irradiation was recorded at position F up to the time when a new IC was incorporated when the minimum dose location changed to D. The maximum dose location is position H. The reference dosimeter used in PfSPZ Vaccine manufacturing runs is located at position B. The relationship between the dose received at B to the dose received at D (or previously, F) and between the dose at B and the dose at H are used to generate the formulae for estimating the doses at positions D/F and H during vaccine production.

Aseptically-reared PfSPZ-infected *Anopheles stephensi* mosquitoes are transferred to Infected Mosquito Transport Containers (IMTC) for aseptic transport to the irradiator. The IMTC consists of a custom designed outer container (OC) fabricated from polycarbonate and composed of a cylinder with a screw-on base and a screw lid that incorporates a filter (**Figure 2**). The IMTC is assembled with the inner container (IC), a modified 1-pint cardboard cylinder, autoclaved, and mosquitoes are aspirated under aseptic conditions directly into the IC from the adult mosquito containers. Each IMTC is sealed inside a sterility maintenance bag (Steris, Erie, PA) which remains in place during irradiation. IMTCs were fabricated with the base able to fit within the circular wall of the irradiator chamber turntable and with a base thickness aimed to position the center of the vertical axis of the IC in the center of the irradiation field in the chamber.

## Radiochromic Film Mapping of the IMTC

The radiation dose received at any point in the irradiator chamber is inversely proportional to the distance from the sources. Thus the dose delivered inside the IMTC will vary both horizontally and vertically. By mapping the radiation field using radiochromic film, the maximum dose and minimum dose locations inside the IMTC can be identified (21). Two sets of radiochromic film mapping experiments were conducted, the first initially after the irradiator was installed and the second following the irradiator upgrade nine years later.

The first determination of the irradiation field was conducted inside the OC of the IMTC using GAFchromic HD-810 film [International Specialty Products (ISP), Wayne, NJ] rated for a dose range of 10-400 Gy. The film was trimmed to fit vertically into the OC, sandwiched between polystyrene plates and sealed in a black polyethylene pouch. Two different OCs were mapped using an irradiation time (provided by JL Shepherd) to target a dose on that particular day of 150 Gy in the center of the OC when the IMTC was placed on the turntable at position 3. The films were removed from their packaging at the National Institute of Standards and Technology (NIST, Gaithersburg, MD), and scanned into a Pharmacia-LKB 2222 UltroScan XL Laser Densitometer at 633 nm with a spot size of 100 μm. Measurements were made by stepping in both dimensions at a resolution of 0.6 mm. The data output was in arbitrary scanning laser densitometer (SLD) units related to optical absorbance. Average SLD values were determined at the film’s center, and the value used to normalize to the whole scan and to express the results in terms of percent increase or decrease relative to the dose at the center.

An additional set of radiochromic film assessments was made following the irradiator upgrade to confirm the distribution of the delivered radiation dose applied to the IC and confirm the minimum and maximum dose locations. Gafchromic Ashland Dose-Map™ film with an upper exposure bound of 50 Gy was sandwiched between Plexiglas sheets and sealed in black polypropylene. This smaller film package was supported inside the IC along the central vertical axis. An alanine transfer dosimeter was also placed on the outside of the OC of the IMTC at location B (**Figure 2**), and the IMTC exposed to a target dose of 50 Gy at the maximum dose location inside the IC.

### Alanine Transfer Dosimeter Mapping of the IMTC

To further characterize the distribution of radiation received by the IMTC and to determine the doses received at the minimum and maximum dose locations when targeting a received minimum dose of 150 Gy, the IC of the IMTC was also mapped using alanine transfer dosimeters (22–25). Alanine dosimeters (NIST High Dose Radiation Service, Gaithersburg, MD) were composed of Plexiglas vials each containing four alanine pellets. The dosimeters were positioned at the locations indicated in **Figure 2**. After irradiation, dosimeters were processed by NIST and the average dose received by the four pellets inside each Plexiglas vial was reported to the nearest whole Gy for that dosimeter.

### Dosimetry to Determine the Irradiation Time for the Target Minimum Dose of 150 Gy

Dosimeters located at the minimum dose location were irradiated on a specific date for times calculated to deliver doses ranging from 120 Gy to 170 Gy. Dosimeters were processed as above and the data for irradiation time and dose received by each dosimeter, were used in a regression analysis, including the upper and lower 95% confidence intervals, to determine the time required to deliver 150 Gy of radiation at the minimum dose location on that date. The value was also used to extrapolate back to the reference date when the irradiator was installed. This experiment was repeated after the irradiator source upgrade.

### Time Table for Irradiation

Two irradiation time tables were constructed spanning 1) the period from initial installation of the irradiator to the upgrade nine years later, and 2) all dates during the subsequent six years. For both timetables the baseline date was defined as the reference date from which to calculate the times to deliver the target minimum dose on all subsequent days according to the equation:


Equation 1 $$t=x*\left(\frac{1}{0.5^{\left(\frac{y}{T}\right)}}\right)$$

where:

t = time in minutes for the day of interest,

x = time in minutes at reference date,

y = number of days since reference date, and

T = ½ life of ^60^Co in days (1925.20 days).

### Use of a Reference Dosimeter and Estimation of the Minimum and maximum doses Delivered to Mosquitoes

The minimum and maximum doses of irradiation received by any mosquito inside the IMTC were estimated from the dose received by a dosimeter attached to the outside of the IMTC at location B (reference location) (**Figure 2**). To determine the formulae for estimating the dose received at the minimum dose location (location D or F) and the maximum dose location (location H) inside the IMTC from the dose received at the reference location, dosimeters were mounted on a cardboard scaffold at the three locations in the IC and at the external reference location.

Three sets of data were collected following installation of the irradiator, three more sets were collected when the irradiator was upgraded, and a final three sets were collected when the original IC was replaced by a new IC of slightly different dimensions. This last data set resulted in the minimum dose location changing from location F to location D (**Figure 2**).

### *In Vitro* Assessment of Attenuation

In addition to dosimetry, the 6-day hepatocyte attenuation assay, a biological measure used to confirm attenuation, was performed using irradiated PfSPZ without cryopreservation (26); the result of this assay, along with the dosimetry data, is incorporated into the lot release certificate of analysis for PfSPZ Vaccine.

### Irradiation Data From PfSPZ Vaccine Manufacture

A manufacturing campaign for PfSPZ Vaccine consists of multiple sequential irradiation runs, generally up to 16. An alanine dosimeter is included at the reference location on the outside of each IMTC for every irradiation run. The irradiation time for any given date is indicated in the irradiation timetables. All dosimeters are processed by NIST. The data for the estimated doses delivered to the minimum dose location were calculated for all runs from the doses reported for the reference dosimeter.

## Results

### Mapping of the Infected Mosquito Transport Container (IMTC) to Determine the Minimum and Maximum Dose Locations

**Figure 3:**
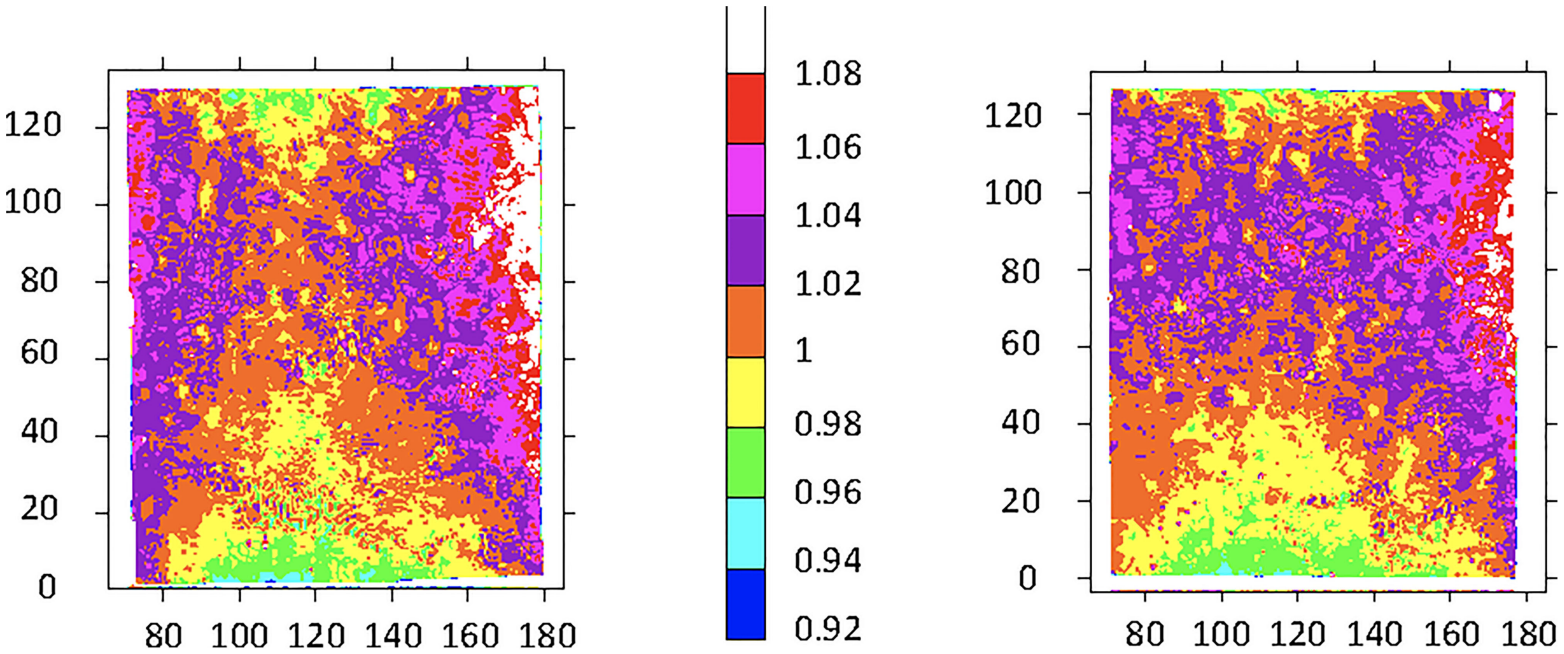
Test film results from two OCs exposed to 150 Gy. The target dose delivered was 150 Gy. The color scale represents proportional arbitrary scanning laser densitometry units normalized to 312 SLD units left, and to 320 SLD units right. The boundary between orange and yellow was assigned a value of 1. Horizontal axis: units in mm relative to the scanner base plate; vertical axis: distance in mm from the bottom of the film/container. See **Figure 2** for the minimum dose locations (position F, bottom center of each film) and maximum dose locations (position H, at the side wall equator of each film).

The purpose of these experiments was to determine the relative radiation dose delivered spatially, which is independent of a particular target dose or dose rate. Three pairs of radiochromic film images were recorded for the original study in the OC of the IMTC. After the first pair of images was obtained, the vertical positioning of the OC was adjusted upwards to improve the vertical gradient of received dose. An additional adjustment to the configuration of the IMTC was made after the second pair of images was obtained; results for the third pair are shown in **Figure 3**. Radiation exposure followed a gradient, with the highest dose received at the vertical midpoint on the side wall decreasing to the center of the OC and decreasing further both upwards and downwards along the central vertical axis. The lowest doses were recorded at the top center and bottom center of the OC.

**Figure 4:**
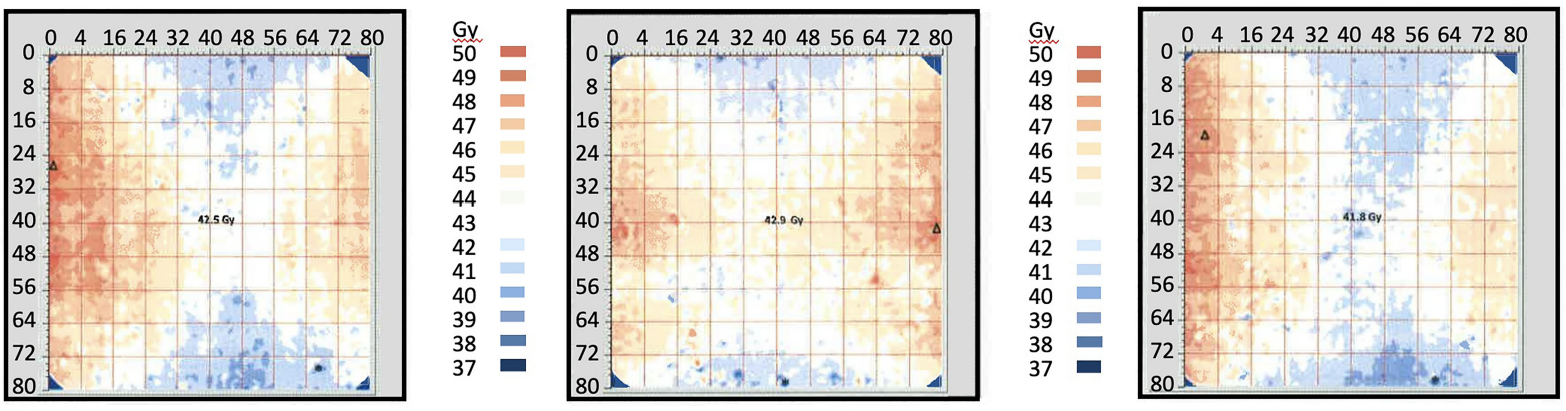
Radiochromic film mapping of the IC of the IMTCs: three runs. The image from run 1 is at left, from run 2 in the center and from run 3 is at right. The dose received at the center of the film (position E) was 42.9 Gy (run 1), 41.8 Gy (run 2) and 42.5 Gy (run 3). See **Table 2** for dose levels recorded for the minimum dose locations (position F, bottom center of each film) and maximum dose locations (position H, at the side wall equator of each film).

Following upgrading of the irradiator, GAF chromic film was provided cut to fit the inside of the IC. For the three films the mean dose ± SD at the minimum dose location (position F) was 38.8 ± 0.64 Gy, and at the maximum dose location (position H) was 47.1 ± 0.25 Gy (**Figure 4**, **Table 1**). The dose uniformity, the ratio of highest dose to lowest dose, (dose at position H/dose at position F) was 1.216 ± 0.014 for this film using a target dose of 50 Gy.

**Table 1:** Radiation doses recorded in the ICs of the IMTCs in the radiochromic film study in **Figure 4**.

Data source	Position in IMTC	Run Number			Mean ± SD
		1	2	3	
		**Radiation dose received (Gy)**		
Alanine dosimeter	B	49.6	49.7	49.4	49.6 ± 0.15
Radiochromic film	E	42.9	41.8	42.5	42.4 ± 0.56
	F	38.3	38.5	39.5	38.8 ± 0.64
	H	46.9	47.1	47.4	47.1 ± 0.25
		**Radiation dose ratio**		
Dose uniformity	H/F	1.225	1.223	1.200	1.216 ± 0.014
Adjustment factor, minimum dose	F/B	0.772	0.775	0.800	0.782 ± 0.015
Adjustment factor, maximum dose	H/B	0.946	0.948	0.960	0.951 ± 0.008

The doses received at the center of each film (location E), at the minimum dose location (location F, up to 2019), and the maximum dose location (location H) are shown together with the doses received by the reference alanine dosimeters at location B. Dose uniformity describes the range of dose between the maximum dose location (H) and the minimum dose location (F) expressed as H/F. The adjustment factors for estimating the minimum dose at F and the maximum dose at H from the dose received by the reference dosimeter at B are also included.

### IMTC (IC) Mapping by Alanine Dosimetry

Experiments were conducted using alanine transfer dosimeters with a dose calculated to deliver 150 Gy to the minimum dose location. The most recent set of these experiments was performed after the changeover to the new IC in the IMTC. These studies used dosimeters placed at the six locations inside the IC (**Figure 2**) to define the minimum and maximum dose locations, and two dosimeters on the exterior of the IMTC at positions A and B (**Figure 2**). In this study, dose uniformity was tighter at 1.09 ± 0.004 (**Table 2**) than seen with GAF chromic film. This alanine dosimeter mapping study also established that the minimum dose was received at location D (154 ± 1.0 Gy) rather than location F (155 ± 1.0 Gy). This change in minimum dose location was a consequence of the changeover to the new IC of the IMTC which was, as indicated above, slightly taller (by 8.7 mm). The position of the base of the IC is fixed, so that the additional height of the IC moved the top of the IC and the minimum dose location higher up the vertical axis of the unit into a lower isodose band. The ratios between the minimum dose and the reference dose (0.834 ± 0.008) and the maximum dose and the reference dose (0.908 ± 0.005) were also established for estimating the minimum and maximum doses delivered during PfSPZ Vaccine production runs. For example, if the dosimeter at the reference location received a dose of 182 Gy, then 151.8 Gy and 165.3 Gy would be received at the minimum and maximum dose locations, respectively, and the dose uniformity (ratio of highest dose to lowest dose), would be 1.089.

**Table 2:** Mapping of the new ICs.

Data source	Position in IMTC	Run Number			Mean ± SD
		1	2	3	
		**Radiation dose received (Gy)**		
Alanine dosimeter	B	184.0	184.0	186.0	184.7 ± 1.2
	D	153.0	155.0	154.0	154.0 ± 1.0
	F	154.0	156.0	155.0	155.0 ± 1.0
	H	167.0	168.0	168.0	167.7 ± 0.6
		**Radiation dose ratio**		
Dose uniformity	H/D	1.0915	1.0839	1.0909	1.0888 ± 0.004
	H/F	1.0844	1.0769	1.0839	1.0817 ± 0.004
Adjustment factor, minimum dose	D/B	0.8315	0.8424	0.8280	0.8340 ± 0.008
	F/B	0.8370	0.8478	0.8333	0.8394 ± 0.008
Adjustment factor, maximum dose	H/B	0.9076	0.9130	0.9032	0.9080 ± 0.005

Irradiation doses received at the two alternative minimum dose locations (positions D and F) and at the maximum dose location (position H) inside the IC of the IMTC, and the dose received at the reference dose location (position B). Also reported are the ratios of the doses received at each location (positions D, F and H) for the three ICs with reference to the doses received at the reference location (B) and the dose uniformity values (H/D and H/F). All runs were performed on 25 July 2019 using an irradiation time of 3.85 minutes to deliver an estimated dose of 150 Gy at position D.

### Dose Titration

**Figure 5:**
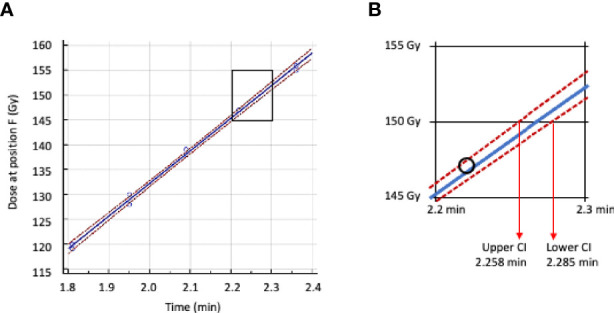
Regression analysis of irradiation time vs dose received at location F (minimum dose location) inside the IMTC. **(A)** Regression line plot in blue, 95% confidence intervals in red. **(B)** Area in A between 145 Gy and 155 Gy enlarged with the lower and upper 95% confidence intervals. The time adopted for calculating the target dose of 150 Gy is 2.285 min.

Dosimeters placed at the minimum dose location in the IC of the IMTC were irradiated for different times to deliver doses between 120 and 170 Gy. Regression analysis of irradiation time vs. dose was used to determine the time to deliver a dose of 150 Gy with lower 95% confidence interval (**Figures 5A, B**). This dose was defined as the target minimum dose and the time to deliver this dose extrapolated from the regression analysis as the time on that date to deliver the target dose of irradiation. This time was then extended to the reference date and that value incorporated as the reference time (x in Equation 1) for constructing the calendar of irradiation timetables.

### Dosimetry for PfSPZ Vaccine Production

The estimated irradiation doses at the minimum dose location (locations F or D) in the IC were calculated using the formulae obtained by dosimetry. Three different sets of conversion factors were used following dosimetry calibration of the irradiator for 1) the period when two sources were active in the irradiator (first 9 years), 2) after the irradiator upgrade when all three sources were active (next 4 years), and 3) after introduction of the new IC (all subsequent times). For all irradiation runs the PfSPZ-infected mosquitoes were irradiated at ambient temperature inside the irradiator chamber which was typically 23°C, a temperature optimum for PfSPZ (26).

For the initial 9-year period the formula for estimating the dose received at location F was x0.8973 and for estimating the maximum dose at location H, the formula used was x0.9471. For the 4 years after the irradiator upgrade the conversion factor used for the dose at location F was x0.845, and for the maximum dose location H, was x0.9141. The current conversion factor used to estimate the minimum dose at location D from the dose received at location B for the period of 2019-present shown in **Table 2** is x0.834, and to convert the dose at location B to the estimated dose at the maximum dose location, location H, the conversion factor is x0.908.

**Figure 6:**
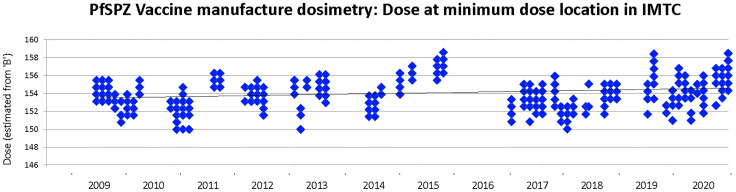
Minimum dose of irradiation received by any mosquito during PfSPZ Vaccine production campaigns between 2009-2020. The target minimum dose is 150 Gy and the minimum acceptable dose is 142.5 Gy. Not all production campaigns were converted into lots released for clinical use, and none of the clinical released lots included PfSPZ irradiated below 150 Gy.

The estimated minimum dose received in each of 587 irradiation events during GMP manufacturing of PfSPZ Vaccine was very consistent (**Figure 6**), ranging from 150.0 Gy to 159.3 Gy, with a mean estimated minimum dose of 154.3 ± 1.77 Gy. The mean estimated maximum dose delivered in all irradiation runs was 166.3 ± 3.65 Gy, (range 155.7 Gy to 175.3 Gy), well below the highest acceptable maximum dose of 190 Gy. The overall dose uniformity was 1.078 ± 0.012.

The viability of PfSPZ in production lots is assessed using a sporozoite membrane integrity assay (SMIA), and is routinely conducted on vaccine bulk product prior to fill-finish. The SMIA results for PfSPZ Vaccine (radiation-attenuated) and PfSPZ Challenge (non-irradiated) have been published previously on several occasions: for example, the viability of PfSPZ after irradiation and prior to cryopreservation was reported as 97% (17) and for non-irradiated PfSPZ prior to cryopreservation the viability has been reported as 98.2% (27).

### Clinical Trials of Radiation-Attenuated PfSPZ in PfSPZ Vaccine

In the 21 clinical trials conducted to date using radiation-attenuated PfSPZ, 1,740 volunteers aged 5 months to 61 years have received 5,648 doses of PfSPZ Vaccine, meaning that >5.3 x 10^9^ irradiated PfSPZ have been administered to human subjects. There have been no breakthrough infections. The 100% infectious dose (ID_100_) for non-irradiated PfSPZ (Sanaria^®^ PfSPZ Challenge (NF54)) administered by direct venous inoculation (DVI) is 3.2 x 10^3^ PfSPZ (27), which has been confirmed in 79 of 79 malaria-naive subjects receiving their first CHMI given by injection (28–31). The highest dose of PfSPZ Vaccine administered has been 2.7 x 10^6^ PfSPZ, which represents 840x the ID_100_ [(32, 33), Sirima et al, submitted for publication]. Overall, the equivalent of more than 1.6 x 10^6^ ID_100_s have been administered without a breakthrough.

## Discussion

We describe here the studies supporting the GMP radiation-attenuation methodology used in the manufacture of PfSPZ Vaccine, a radiation-attenuated, purified and cryopreserved, metabolically-active, non-replicating, whole sporozoite vaccine against malaria which has demonstrated unparalelled efficacy, safety and tolerability. In the 21 clinical trials conducted to date, three trials have shown 100% protection against homologous controlled human malaria infection (CHMI) (17, 19, 20).

Some of the initial concerns considered during development of a radiation-attenuated malaria vaccine were that attenuation by irradiation would be difficult to manage, that the parasites would have the potential to cause infections because of inadequate attenuation, or would cause an inferior and non-protective immune response due to over attenuation. None of these three scenarios has occurred.

Although the precise mechanisms whereby irradiation prevents replication is not understood, it is likely that damage to DNA results in multiple redundant defects providing a high level of assurance that no individual parasite would be capable of replication. Parasite genes that do appear to be downregulated following irradiation include those for DNA repair (34). Thus, with over 5.3 x 10^9^ PfSPZ irradiated and subsequently administered to humans, not one has broken through. As the data presented here demonstrate, the manufacturing process for PfSPZ Vaccine maintains a tight control over the attenuation process.

PfSPZ administered directly by the bite of mosquitoes subjected to 150 Gy did not lead to breakthrough infections whereas PfSPZ exposed to a dose 120 Gy PfSPZ were not fully attenuated and breakthrough infections occurred (11, 12). In the *in vitro* 6-day hepatocyte attenuation assay, liver stage parasites develop from PfSPZ subjected to 120 Gy, but not after an irradiation dose of 142.5 Gy (data to be published separately). For the rodent malaria parasite, *P. yoelii* (Py), which has a lower tolerance to irradiation than Pf, the minimum predicted dose to achieve full attenuation in mice is 92.4 Gy (35). At a radiation dose of 100 Gy, PySPZ were fully attenuated; injection of 1x10^5^ irradiated PySPZ to each of 41 mice failed to lead to infection, compared to a dose of just 2.78 non-irradiated PySPZ that infected 50% of the mice (33). The minimum attenuating dose for *P. berghei* (Pb) also appears to be 100 Gy (10), although most studies of immunity stimulated by PbSPZ utilize 120-150 Gy (36, 37).

For manufacturing PfSPZ Vaccine, we chose a target minimum dose of 150 Gy with an acceptable minimum dose of 142.5 Gy (5% below the target dose) and a maximum dose of 190 Gy. If over-attenuated, the PfSPZ could potentially lose their ability to stimulate a protective immune response in the liver. For the rodent parasite PySPZ, doubling the attenuating dose (i.e. to 200 Gy) does not lead to a diminution of protection. However, we wanted to provide a tighter range for the PfSPZ so selected, in the absence of any available data, a dose of 190 Gy as the upper threshold for acceptance. In practice, as indicated here, the highest level of irradiation for the PfSPZ has been considerably lower with a range maximum of 175.3 Gy, which is well below the acceptable maximum dose. Overall, the mean estimated minimum dose was 154.3 Gy and the mean estimated maximum dose was 166.3 Gy for vaccine lots.

Irradiation using ^60^Co is a physical process. When incorporating the defined half life decay of the sources (Equation 1) it should be possible to deliver a precise dose of radiation and for there to be no meaningful variability between runs on the same day or between runs on different days. However, the irradiator has moving parts with some inertia or variability – these include the rotation of the turntable in the irradiator chamber, the position of the reference dosimeter relative to the sources when they are activated during a run, and the speed with which the source rods are elevated by air pressure from the compressor. There are uncertainties in the alanine processing methodology to which NIST assigns a value of ±1.8%. Together some of these factors may account for a portion of the overall ±3.1% (150-159.3 Gy) variability seen between runs (**Figure 6**).

^60^Co irradiation has proven to be a robust, repeatable and reliable process, but has drawbacks because of attendant security issues which significantly increase the cost, including running personnel background checks, training and certification, and security of controlled access and monitoring. These are fixed costs that would be diluted by scaling up production, however, because of the half life decay, the sources deplete over time and have to be replenished. Our irradiator has undergone one upgrading event at considerable cost. In the future it is likely that ^60^Co irradiation will be replaced by X-ray irradiation, and Sanaria has begun to explore the process of making this transition.

## Data Availability Statement

The original contributions presented in the study are included in the article. Further inquiries can be directed to the corresponding author.

## Author Contributions

Conceptualization: EJ and SH. Methodology: EJ, SM, JO, BS, AE, TL, ML, PB, TR, SC, AG, and TM. Funding acquisition: EJ and SH. Project administration: EJ and SH. Supervision: EJ and SH. Writing – original draft: EJ. Writing – review & editing: EJ, SH, PB, and TR. All authors contributed to the article and approved the submitted version.

## Funding

The work was supported by components of several Small Business Research Innovation awards to SH from the National Institute of Allergy and Infectious Disease of NIH, principally 43AI058499 and 44AI058499.

## Conflict of Interest

All authors are employed by Sanaria Inc.

## Publisher’s Note

All claims expressed in this article are solely those of the authors and do not necessarily represent those of their affiliated organizations, or those of the publisher, the editors and the reviewers. Any product that may be evaluated in this article, or claim that may be made by its manufacturer, is not guaranteed or endorsed by the publisher.

## Footnotes

1
https://www.vmd.defra.gov.uk/productinformationdatabase/files/QRD_Documents/QRD-Auth_1189554.PDF.
